# Supplementary material for: Modeling glioblastoma heterogeneity as a dynamic network of cell states
Source: Mol Syst Biol. 2021 Sep 16;17(9):e10105. doi: 10.15252/msb.202010105 (PMC8444284; doi:10.15252/msb.202010105)
Supplement: Supplementary file 6 — Source Data for Figure 5 [file MSB-17-e10105-s004.zip › Figure5A_sourcedata/GSEA_3017/nosigdb_stateD.GseaPreranked.1622795010397/FELDMAN_2020_G2-M.html]

Details for gene set FELDMAN\_2020\_G2-M[GSEA]

|  || Dataset | state23017 |
| Phenotype | NoPhenotypeAvailable |
| Upregulated in class | na\_neg |
| GeneSet | FELDMAN\_2020\_G2-M |
| Enrichment Score (ES) | -0.51812905 |
| Normalized Enrichment Score (NES) | -2.9325144 |
| Nominal p-value | 0.0 |
| FDR q-value | 0.0 |
| FWER p-Value | 0.0 |
Table: GSEA Results Summary

  

Fig 1: Enrichment plot: FELDMAN\_2020\_G2-M      
 Profile of the Running ES Score & Positions of GeneSet Members on the Rank Ordered List

  

| PROBE | GENE SYMBOL | GENE\_TITLE | RANK IN GENE LIST | RANK METRIC SCORE | RUNNING ES | CORE ENRICHMENT || 1 | PTTG1 |  |  | 14 | 0.490 | -0.0048 | No |
| 2 | RAB13 |  |  | 43 | 0.367 | -0.0232 | No |
| 3 | BTG1 |  |  | 50 | 0.350 | -0.0232 | No |
| 4 | HES6 |  |  | 60 | 0.336 | -0.0260 | No |
| 5 | HMGB3 |  |  | 80 | 0.311 | -0.0376 | No |
| 6 | CALM3 |  |  | 82 | 0.308 | -0.0341 | No |
| 7 | SOX4 |  |  | 91 | 0.298 | -0.0366 | No |
| 8 | LDHA |  |  | 92 | 0.297 | -0.0323 | No |
| 9 | TROAP |  |  | 109 | 0.283 | -0.0418 | No |
| 10 | LSM5 |  |  | 123 | 0.274 | -0.0488 | No |
| 11 | BIRC5 |  |  | 130 | 0.271 | -0.0500 | No |
| 12 | MKI67 |  |  | 206 | -0.254 | -0.1096 | No |
| 13 | PLD3 |  |  | 240 | -0.258 | -0.1338 | No |
| 14 | FTL |  |  | 271 | -0.261 | -0.1554 | No |
| 15 | TRIP13 |  |  | 294 | -0.263 | -0.1702 | No |
| 16 | GPM6B |  |  | 314 | -0.265 | -0.1824 | No |
| 17 | HMMR |  |  | 319 | -0.266 | -0.1820 | No |
| 18 | EXOSC8 |  |  | 347 | -0.268 | -0.2009 | No |
| 19 | PRR11 |  |  | 361 | -0.269 | -0.2081 | No |
| 20 | ISCA2 |  |  | 386 | -0.272 | -0.2244 | No |
| 21 | TUBB |  |  | 410 | -0.274 | -0.2399 | No |
| 22 | GLIPR2 |  |  | 411 | -0.275 | -0.2360 | No |
| 23 | CDC25B |  |  | 472 | -0.282 | -0.2825 | No |
| 24 | FOXM1 |  |  | 478 | -0.282 | -0.2827 | No |
| 25 | ECT2 |  |  | 481 | -0.283 | -0.2804 | No |
| 26 | RHNO1 |  |  | 523 | -0.288 | -0.3109 | No |
| 27 | PSRC1 |  |  | 574 | -0.295 | -0.3488 | No |
| 28 | SPAG5 |  |  | 605 | -0.301 | -0.3698 | No |
| 29 | PRC1 |  |  | 645 | -0.310 | -0.3983 | No |
| 30 | PRPSAP1 |  |  | 660 | -0.312 | -0.4056 | No |
| 31 | RAD21 |  |  | 674 | -0.314 | -0.4121 | No |
| 32 | TMPO |  |  | 688 | -0.317 | -0.4185 | No |
| 33 | KNSTRN |  |  | 690 | -0.317 | -0.4149 | No |
| 34 | S100B |  |  | 705 | -0.320 | -0.4221 | No |
| 35 | KIF4A |  |  | 706 | -0.320 | -0.4175 | No |
| 36 | MPHOSPH9 |  |  | 713 | -0.321 | -0.4180 | No |
| 37 | IGFBP7 |  |  | 733 | -0.326 | -0.4294 | No |
| 38 | SPC24 |  |  | 747 | -0.330 | -0.4356 | No |
| 39 | CDC20 |  |  | 793 | -0.341 | -0.4687 | No |
| 40 | H2AFX |  |  | 810 | -0.347 | -0.4772 | No |
| 41 | NCAPD2 |  |  | 811 | -0.347 | -0.4723 | No |
| 42 | CENPM |  |  | 818 | -0.349 | -0.4724 | No |
| 43 | MAD2L1 |  |  | 819 | -0.350 | -0.4674 | No |
| 44 | BUB3 |  |  | 826 | -0.351 | -0.4674 | No |
| 45 | SHCBP1 |  |  | 844 | -0.356 | -0.4767 | No |
| 46 | GAS2L3 |  |  | 856 | -0.359 | -0.4808 | No |
| 47 | NEK2 |  |  | 861 | -0.359 | -0.4791 | No |
| 48 | MEST |  |  | 864 | -0.360 | -0.4756 | No |
| 49 | CKAP2 |  |  | 900 | -0.375 | -0.4998 | No |
| 50 | TK1 |  |  | 902 | -0.375 | -0.4953 | No |
| 51 | TUBB4B |  |  | 916 | -0.380 | -0.5008 | No |
| 52 | TUBG1 |  |  | 917 | -0.381 | -0.4954 | No |
| 53 | DEPDC1B |  |  | 923 | -0.383 | -0.4942 | No |
| 54 | DIAPH3 |  |  | 943 | -0.390 | -0.5046 | No |
| 55 | TMEM237 |  |  | 953 | -0.396 | -0.5066 | No |
| 56 | DCBLD2 |  |  | 957 | -0.397 | -0.5034 | No |
| 57 | SKA3 |  |  | 965 | -0.399 | -0.5036 | No |
| 58 | RTKN2 |  |  | 973 | -0.403 | -0.5038 | No |
| 59 | FILIP1L |  |  | 991 | -0.411 | -0.5123 | Yes |
| 60 | SPDL1 |  |  | 995 | -0.412 | -0.5089 | Yes |
| 61 | FAM83D |  |  | 998 | -0.413 | -0.5047 | Yes |
| 62 | KIF11 |  |  | 1003 | -0.416 | -0.5022 | Yes |
| 63 | CCDC34 |  |  | 1013 | -0.421 | -0.5037 | Yes |
| 64 | CMC2 |  |  | 1017 | -0.425 | -0.5002 | Yes |
| 65 | MIS18A |  |  | 1020 | -0.427 | -0.4958 | Yes |
| 66 | TPX2 |  |  | 1034 | -0.437 | -0.5005 | Yes |
| 67 | KIF22 |  |  | 1051 | -0.446 | -0.5077 | Yes |
| 68 | SMC2 |  |  | 1062 | -0.452 | -0.5097 | Yes |
| 69 | CDKN2C |  |  | 1067 | -0.454 | -0.5065 | Yes |
| 70 | DEPDC1 |  |  | 1074 | -0.457 | -0.5051 | Yes |
| 71 | PLK1 |  |  | 1082 | -0.460 | -0.5044 | Yes |
| 72 | ZWINT |  |  | 1099 | -0.467 | -0.5113 | Yes |
| 73 | UBC |  |  | 1102 | -0.469 | -0.5063 | Yes |
| 74 | SAT1 |  |  | 1109 | -0.473 | -0.5046 | Yes |
| 75 | BUB1B |  |  | 1111 | -0.477 | -0.4986 | Yes |
| 76 | RACGAP1 |  |  | 1112 | -0.479 | -0.4918 | Yes |
| 77 | TTK |  |  | 1119 | -0.487 | -0.4899 | Yes |
| 78 | SPARC |  |  | 1127 | -0.493 | -0.4888 | Yes |
| 79 | TTYH1 |  |  | 1144 | -0.505 | -0.4950 | Yes |
| 80 | CKAP2L |  |  | 1147 | -0.508 | -0.4895 | Yes |
| 81 | TMEM106C |  |  | 1148 | -0.509 | -0.4822 | Yes |
| 82 | FTH1 |  |  | 1152 | -0.514 | -0.4774 | Yes |
| 83 | MXD3 |  |  | 1153 | -0.514 | -0.4701 | Yes |
| 84 | NUSAP1 |  |  | 1154 | -0.515 | -0.4627 | Yes |
| 85 | GMNN |  |  | 1155 | -0.516 | -0.4554 | Yes |
| 86 | DBF4 |  |  | 1157 | -0.520 | -0.4488 | Yes |
| 87 | MIS18BP1 |  |  | 1162 | -0.524 | -0.4447 | Yes |
| 88 | KIF14 |  |  | 1168 | -0.535 | -0.4413 | Yes |
| 89 | KIF2C |  |  | 1183 | -0.558 | -0.4451 | Yes |
| 90 | CDCA3 |  |  | 1187 | -0.563 | -0.4396 | Yes |
| 91 | ASPM |  |  | 1189 | -0.567 | -0.4324 | Yes |
| 92 | FABP7 |  |  | 1190 | -0.568 | -0.4243 | Yes |
| 93 | NUF2 |  |  | 1194 | -0.574 | -0.4186 | Yes |
| 94 | CKS1B |  |  | 1198 | -0.577 | -0.4129 | Yes |
| 95 | CLU |  |  | 1207 | -0.592 | -0.4112 | Yes |
| 96 | CST3 |  |  | 1209 | -0.595 | -0.4036 | Yes |
| 97 | GTSE1 |  |  | 1210 | -0.602 | -0.3950 | Yes |
| 98 | BEX1 |  |  | 1216 | -0.615 | -0.3904 | Yes |
| 99 | SERPINE1 |  |  | 1218 | -0.617 | -0.3825 | Yes |
| 100 | CENPK |  |  | 1220 | -0.619 | -0.3745 | Yes |
| 101 | SMC4 |  |  | 1224 | -0.638 | -0.3679 | Yes |
| 102 | CKS2 |  |  | 1226 | -0.641 | -0.3596 | Yes |
| 103 | ANLN |  |  | 1229 | -0.646 | -0.3521 | Yes |
| 104 | KIF18A |  |  | 1231 | -0.649 | -0.3437 | Yes |
| 105 | CTGF |  |  | 1234 | -0.654 | -0.3360 | Yes |
| 106 | CDCA8 |  |  | 1239 | -0.669 | -0.3298 | Yes |
| 107 | CENPA |  |  | 1243 | -0.679 | -0.3227 | Yes |
| 108 | AURKB |  |  | 1250 | -0.700 | -0.3178 | Yes |
| 109 | MELK |  |  | 1251 | -0.701 | -0.3078 | Yes |
| 110 | CCNA2 |  |  | 1255 | -0.716 | -0.3001 | Yes |
| 111 | UBE2T |  |  | 1256 | -0.719 | -0.2898 | Yes |
| 112 | CDCA2 |  |  | 1258 | -0.727 | -0.2803 | Yes |
| 113 | ARHGAP11A |  |  | 1262 | -0.735 | -0.2724 | Yes |
| 114 | HMGB2 |  |  | 1263 | -0.736 | -0.2619 | Yes |
| 115 | KIF23 |  |  | 1265 | -0.741 | -0.2521 | Yes |
| 116 | KIFC1 |  |  | 1267 | -0.748 | -0.2423 | Yes |
| 117 | TACC3 |  |  | 1268 | -0.751 | -0.2316 | Yes |
| 118 | AURKA |  |  | 1271 | -0.763 | -0.2224 | Yes |
| 119 | NDC80 |  |  | 1278 | -0.810 | -0.2159 | Yes |
| 120 | IFITM3 |  |  | 1279 | -0.812 | -0.2043 | Yes |
| 121 | ESCO2 |  |  | 1283 | -0.846 | -0.1948 | Yes |
| 122 | CALM2 |  |  | 1286 | -0.878 | -0.1840 | Yes |
| 123 | MALAT1 |  |  | 1289 | -0.884 | -0.1730 | Yes |
| 124 | HJURP |  |  | 1290 | -0.897 | -0.1602 | Yes |
| 125 | SPC25 |  |  | 1294 | -0.932 | -0.1495 | Yes |
| 126 | RRM2 |  |  | 1295 | -0.933 | -0.1362 | Yes |
| 127 | KPNA2 |  |  | 1297 | -0.972 | -0.1231 | Yes |
| 128 | PIF1 |  |  | 1299 | -0.987 | -0.1099 | Yes |
| 129 | FBXO5 |  |  | 1301 | -0.992 | -0.0966 | Yes |
| 130 | NCAPG |  |  | 1303 | -1.015 | -0.0830 | Yes |
| 131 | CDKN1A |  |  | 1306 | -1.046 | -0.0697 | Yes |
| 132 | IGFBP5 |  |  | 1307 | -1.052 | -0.0547 | Yes |
| 133 | CDK1 |  |  | 1311 | -1.433 | -0.0368 | Yes |
| 134 | UBE2C |  |  | 1312 | -1.466 | -0.0159 | Yes |
| 135 | TOP2A |  |  | 1313 | -1.528 | 0.0059 | Yes |
Table: GSEA details [plain text format]

  

Fig 2: FELDMAN\_2020\_G2-M: Random ES distribution      
 Gene set null distribution of ES for **FELDMAN\_2020\_G2-M**
